# Supplementary material for: Predicting visual function by interpreting a neuronal wiring diagram
Source: Nature. 2024 Oct 2;634(8032):113–23. doi: 10.1038/s41586-024-07953-5 (PMC11446822; doi:10.1038/s41586-024-07953-5)
Supplement: Supplementary file 6 — CRF and ERF predictions for individual TmY4 and TmY9 cells. Analogous to Supplementary Data 3, but for TmY target types. Shown are the top four monosynaptic pathways, the strongest pathway passing through each of the top ten intermediary types (ranking from Extended Data Fig. 7), and the trisynaptic pathway Tm1–TmY–Dm3–TmY (see the section entitled Prediction of spatial normalization). [file 41586_2024_7953_MOESM6_ESM.zip › DataS4/TmY9q/720575940617890777.pdf]

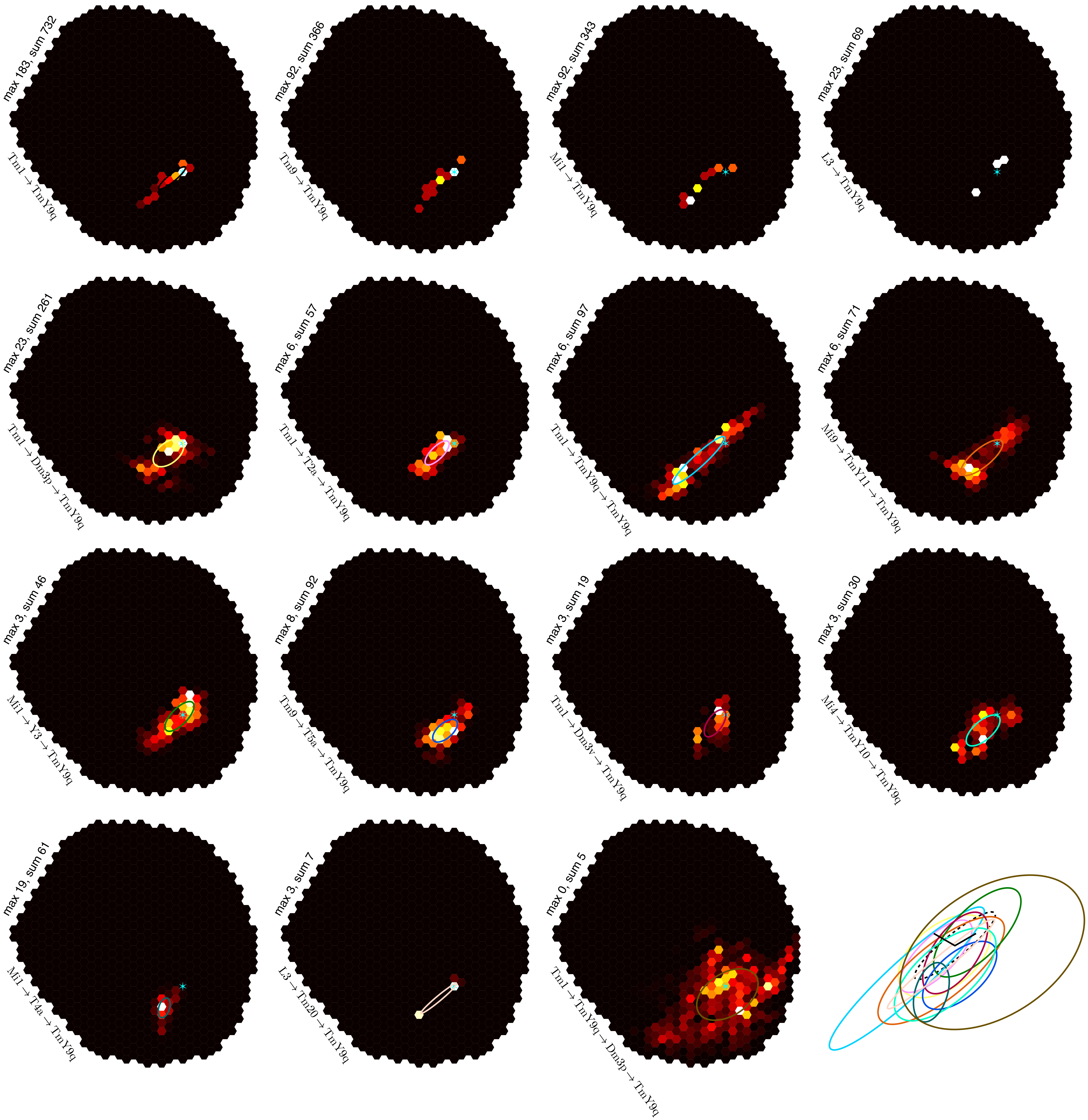
max 183, sum 732
Tm1 → TmY9q
max 92, sum 366
Tm9 → TmY9q
max 92, sum 343
Mi1 → TmY9q
max 23, sum 69
L3 → TmY9q
max 23, sum 261
Tm1 → Dm3p → TmY9q
max 6, sum 57
Tm1 → T2a → TmY9q
max 6, sum 97
Tm1 → TmY9q → TmY9q
max 6, sum 71
Mi9 → TmY11 → TmY9q
max 3, sum 46
Mi1 → Y3 → TmY9q
max 8, sum 92
Tm9 → T5a → TmY9q
max 3, sum 19
Tm1 → Dm3v → TmY9q
max 3, sum 30
Mi4 → TmY10 → TmY9q
max 19, sum 61
Mi1 → T4a → TmY9q
max 3, sum 7
L3 → Tm20 → TmY9q
max 0, sum 5
Tm1 → TmY9q → Dm3p → TmY9q
